# Optimization of process parameters for enhanced production of Jamun juice using Pectinase (*Aspergillus aculeatus)* enzyme and its characterization

**DOI:** 10.1007/s13205-016-0561-0

**Published:** 2016-11-12

**Authors:** Payel Ghosh, Rama Chandra Pradhan, Sabyasachi Mishra

**Affiliations:** Department of Food Process Engineering, National Institute of Technology, Rourkela, Rourkela, 769008 Odisha India

**Keywords:** Jamun fruits, Enzymatic extraction, *Aspergillus aculeatus*, Optimization, Box-Behnken design

## Abstract

Jamun fruit comprises of seed and thick pulp. The pectin–protein bond of the thick pulp creates difficulty in making juice. Clear Jamun juice is not available in the market, so there is a need for extraction of juice with maximum yield. The goal of this research is to obtain high yield of clarified juice with the help of Pectinase (*Aspergillus aculeatus*) enzyme. The study was conducted at different enzyme concentration (0.01–0.1%), time duration (40–120 min), and temperature (30–50 °C). Various physical and chemical parameters, such as yield, turbidity, viscosity, clarity, colour, polyphenol, protein, TSS, and total solid, were measured as dependent variables. Process optimization has been done using Box–Behnken design. Optimization has been done for maximum yield percentage, L* value, a* value, protein, and polyphenol content, and minimum values for turbidity, viscosity, clarity, b* value, TSS, and total solid content. The suggested parameters for extraction of juice were at 0.05% enzyme concentration at 44 °C for 80 min. In a large-scale production, extraction of juice by Pectinase (*Aspergillus aculeatus*) has a significance importance due to its high yield as well as nutritional values.

## Introduction

Fruits are known for their excellent antioxidant properties, therapeutic value, and nutrient enrichment. Jamun (Indian Blackberry) is a tropical natural product, with its antidiabetic properties, which helps in the transformation of starch or sugar into vitality. It is rich in iron and subsequently helps in the purification of blood (Sehwag and Das 2015; Mahesh and Satish 2008; Jabeen and Javaid 2010). Preservation of fresh produced samples over long time is energy consuming and expensive. After harvest, the nutritional and quality attributes of fresh juicy fruit, such as Jamun, get deteriorate as it comes in contact with surrounding of physical and microbial environment. Low yield and low extraction efficiency of Jamun juice have been attributed to chemical structure of the fruit system. Exploration of the structural make up of Jamun fruit has indicated that the presence of higher pectin and tannin content and storage linkage between protein and pectin molecules has resulted in hindrance for extraction efficiency. In case of perishable fruit system, such as Jamun, there is a need for the development of novel strategies for proper processing to get higher juice yield so as to achieve continuous supply. Meanwhile, the application of enzymes for enhancing the yield and extraction efficiency has been given wide range of commercial importance.

In the beverage industry, enzymatic juice extraction process followed by clarification and membrane separation is considered as an advanced step for concentration of juice. However, a very few studies have been done on clarification of natural product with enzymatic pre-treatment particularly for tropical fruit juices (Rai et al. 2004; Sin et al. 2006). In the process of enzymatic pre-treatment for juice clarification, hydrolysis of pectic substances is impacted by few components, for example, enzyme concentration, incubation time, and incubation temperature (Rai et al. 2004; Sin et al. 2006).

In case of fruit juice depectinization and to increase the product yield, several enzymes have been used as a pre-treatment. Several studies have reported on pectinases enzyme for depectinization which could effectively clarify the fruit juices (Tastan and Baysal 2015; Domingues et al. 2012; Tu et al. 2013; Maktouf et al. 2014). A particular enzyme with various strains also acts differently. For example, pectinase obtained from *Aspergillus Niger* will act on a biological system in a certain manner where pectinase obtained from *Aspergillus aculeatus* will work in differently. Therefore, many studies with different strains also reported for various fruits (Ghosh et al. 2016; Sharma et al. 2015; Tapre and Jain 2014; Landbo et al. 2007; Pinelo et al. 2010; Lee et al. 2006; Landbo and Meyer 2004; Sandri et al. 2014).

Response surface Methodology (RSM) is a statistical and mathematical tool valuable for breaking down the total impacts of several independent variables and responses (Box and Draper 1987). One of the types for RSM is known as Box–Behnken design (does not contain an embedded factorial or fractional factorial design) which do not have axial points, thus, can be sure that all design points fall within your safe operating zone. Box–Behnken designs also ensure that all factors are not set at their high levels at the same time. This procedure is more functional contrasted with hypothetical models. It emerges from trial approach which incorporates intelligent impacts of the variables, and inevitably, it portrays the general impacts of the parameters on the procedure (Bas and Boyaci 2007). The most ideal approach to picture the impact of the independent variables on the reliant ones is to draw response surface plots of the model. In this manner, a response surface trial plan was esteemed to be most suitable method for the present examination.

The objectives of this study is to optimize the enzyme concentration, incubation time, and temperature for juice extraction and clarification from Jamun fruit with the help of commercial pectinase (*Aspergillus aculeatus*) using the response surface methodology (RSM). The second objective is to characterise the physico-chemical properties, such as viscosity, turbidity, yield, clarity, colour, total polyphenol, protein concentration, TSS, and total solid in the fresh product, as well as the treated samples.

## Materials and methods

### Jamun fruit sample

Fresh ripen Jamun (locally called as *Ram Jamun*) was obtained from Rourkela, Odisha, India. Due to high perishable nature of Jamun fruits, the samples were manually washed (water with 2% CaCl_2_), cleaned, and then packed in polythene bags. The cleaned samples were stored at −20 ± 1 °C for further study (Benherlal 2010).

### Pectinase enzyme and chemicals

The enzyme pectinase (Commercial Name: Pectinex Ultra SPL; activity 3800 units/ml) from *Aspergillus aculeatus,* and Folin–Ciocalteu reagent was procured from Sigma-Aldrich, Bangalore, India. Hydrazine sulphate, potassium sodium tartrate, and hexamine LR grade were purchased from HiMediaPvt. Ltd., Mumbai, India. Copper (II) sulphate pentahydrate was collected from Merck, Mumbai, India. Bovine serum albumin (BSA) and Gallic acid standard were purchased from Otto Chemie Pvt. Ltd, Mumbai, India.

### Method of Jamun juice extraction

The cleaned Jamun samples from deep freezer were taken out and washed and clean to avoid microbial contaminations, and other foreign particles, if any. Then, the samples were allowed to warm up at room temperature for 3 h. To avoid bitterness of juice, seeds were manually removed from the samples. Collected pulp of the fruit sample then mixed using a grinder (Bajaj Mixer, India). Afterwards, the grinded pulp was poured in beakers. As per the experimental design, the pectinase enzyme was added with the mentioned temperature and time.

### Enzymatic treatment

As mentioned in Table 1 (experimental design), 100 g of homogenised pulp was treated with enzyme. The independent parameters, such as incubation temperature *X*_1_ (30–50 °C), enzyme concentration, *X*_2_ (0.01–0.1 w/v, %), and incubation time, *X*_3_ (40–120 min), were considered. An incubated orbital shaker (REMI Laboratory Instrument, Mumbai, India) at 120 rpm was used for vigorous mixing of enzymatic treated pulp at particular temperature and time. Then, the suspension was kept at −2 °C for 5 min for inactivation of the enzyme (Molinari and Silva 1997; Sandri et al. 2011). A muslin cloth was used to filtrate the juice. The filtrate was taken for further analysis of different physico-chemical properties. One sample was kept without any enzymatic treatment, known as control sample.

**Table 1:** Properties of raw Jamun juice without enzymatic treatment

Property	Yield (%)	Viscosity (mPaS)	Turbidity (NTU)	Clarity (%T)	*L*	a*	b*	Protein (mg/g)	Polyphenol (mg GAE/g)	TSS (°B)	Total solid (%)
Raw Juice	65	1.21	116	0.165	5.76	2.65	−1.61	132.70	89.08	13	12.94

### Characterisation of juice

Physical parameters, such as viscosity, turbidity, yield, clarity, and colour, and chemical parameters, such as protein, polyphenol, TSS, and total solid, were determined as response parameters. A viscometer and a digital turbidity-meter were used to measure the viscosity and turbidity of juice, respectively (Sin et al. 2006). The treated pulp was passed through muslin cloth (Shahnawaz and Sheikh 2011). The percent juice content of Jamun was given as:

$${\text{Percentage Juice content}} = \frac{{{\text{Volume of filtrate }}\left( {\text{ml}} \right)}}{{{\text{Weight of the pulp }}\left( {\text{g}} \right)}} \times 100.$$

Clarity of the juice was determined by transmittance (%T) at 660 nm (Rai et al. 2006) and total phenolic substance was measured by the Folin–Ciocalteu method at 650 nm (% absorbance) (Singleton et al. 1999) using a spectrophotometer (Systronics India Ltd). A Hunter colorimeter (Colorflex EZ, USA) was used for measurements of juice colour {L* (brightness/darkness), a*(redness/greenness), b* (yellowness/blueness)} (Rai et al. 2006). Protein concentration was measured with Bovine Serum Albumin (BSA) as the standard according to method described by Lowry et al. (1951). An Abbe-type refractometer (°B) was used to measure the total soluble sugar (TSS) in the sample. Total solid was measured by weight difference (Sagu et al. 2014).

### Experimental setup

Box–Behnken Design was considered to optimise the treatment and extraction conditions for Jamun juice. The Design Expert Software (Version 8.0.7.1) was used for optimisation of independent variables.

For the enzymatic treatment of juice, the key elements are incubation temperature, enzyme concentration, and incubation time, which coded as X1, X2, and X3, respectively. Box-Behnken Design setup was taken to analyse on the combined impact of these three variables. Total 17 experiments with 5 replications at the center point were designed through software. Tables 2 and 3 show the real and coded values of the experiments, respectively. The effects of variables in terms of linear, quadratic, and interaction terms can be explain by mathematical model as shown below:

$$Y \, = \, b_{0} + \, \sum \, b_{1} X_{1} + \, \sum \, b_{11} X_{1}^{2} + \, \sum \, b_{12} X_{1} X_{2}$$

where *Y* the experimental responses; *X*_1_ and *X*_2_ the levels of variables; *b*_0_ the constant; *b*_1_ the linear coefficient; *b*_11_ the quadratic term; and *b*_12_ the coefficient of the interaction terms.

**Table 2:** Box–Behnken design: real variables and experimental responses (physical parameters)

Exp. no.	Real variables			Experimental responses													
	X_1_ (°C)	X_2_ (%)	X_3_ (min)	Yield (%)		Clarity (%T)		Turbidity (NTU)		Viscosity (mPaS)		L* value		a* value		b* value	
				Act	Pre	Act	Pre	Act	Pre	Act	Pre	Act	Pre	Act	Pre	Act	Pre
1	30	0.01	80	76	75.75	78.33	76.84	14.9	14.70	1.086	1.08	1.17	1.11	0.81	0.83	−0.37	−0.32
2	50	0.01	80	77	76.75	66.40	67.99	46.8	46.74	1.092	1.09	0.53	0.45	1.11	1.11	−0.20	−0.19
3	30	0.10	80	78	78.25	72.97	71.38	20.65	20.71	1.100	1.11	0.68	0.77	1.01	1.01	−0.18	−0.19
4	50	0.10	80	79	79.25	68.63	70.13	45.30	45.50	1.077	1.08	0.55	0.61	1.02	1.00	−0.21	−0.27
5	30	0.06	40	79	79.50	68.60	70.70	19.65	19.61	1.086	1.10	0.93	0.92	0.93	0.90	−0.16	−0.18
6	50	0.06	40	81	81.50	70.33	69.15	46.80	46.62	1.071	1.08	0.55	0.56	1.15	1.14	−0.18	−0.16
7	30	0.06	120	80	79.50	74.30	75.48	18.95	19.13	1.100	1.09	1.00	0.99	0.98	0.99	−0.19	−0.21
8	50	0.06	120	80	79.50	68.83	66.93	48.90	48.94	1.100	1.09	0.52	0.53	0.99	1.03	−0.21	−0.19
9	40	0.01	40	82	81.75	77.30	76.89	13.50	13.74	1.071	1.07	0.89	0.96	0.69	0.71	−0.15	−0.18
10	40	0.10	40	85	84.25	77.13	76.82	12.90	12.88	1.086	1.07	0.92	0.84	0.64	0.67	−0.16	−0.13
11	40	0.01	120	80	80.75	79.43	79.75	11.40	11.42	1.041	1.06	0.87	0.95	0.66	0.62	−0.15	−0.18
12	40	0.10	120	83	83.25	76.10	76.51	17.30	17.06	1.071	1.08	0.97	0.89	0.75	0.73	−0.21	−0.18
13	40	0.06	80	74	73.80	29.47	29.29	32.10	32.36	1.101	1.10	2.13	2.31	3.22	3.21	−1.40	−1.21
14	40	0.06	80	74	73.80	29.43	29.29	32.40	32.36	1.100	1.10	2.18	2.31	3.24	3.21	−1.15	−1.21
15	40	0.06	80	74	73.80	29.03	29.29	32.10	32.36	1.086	1.10	2.51	2.31	3.13	3.21	−1.10	−1.21
16	40	0.06	80	74	73.80	29.33	29.29	32.60	32.36	1.100	1.10	2.54	2.31	3.24	3.21	−1.27	−1.21
17	40	0.06	80	73	73.80	29.17	29.29	32.60	32.36	1.100	1.10	2.19	2.31	3.21	3.21	−1.13	−1.21

**Table 3:** Box–Behnken design: real variables and experimental responses (chemical parameters)

Exp. no.	Coded variables			Experimental responses							
	*x*_1_	*x*_2_	*x*_3_	Protein (mg/g)		Polyphenol (mg GAE/g)		TSS (°B)		Total solid (%)	
				Act	Pre	Act	Pre	Act	Pre	Act	Pre
1	−1	−1	0	368.02	375.47	111.34	110.08	13.00	13.02	15.64	15.59
2	1	−1	0	139.40	142.25	130.58	128.68	13.20	13.23	15.96	15.44
3	−1	1	0	394.99	392.14	119.25	121.14	13.30	13.17	15.42	15.45
4	1	1	0	179.19	171.74	121.92	123.18	13.10	13.17	15.64	15.69
5	−1	0	−1	409.30	403.89	113.81	111.68	13.20	13.15	14.66	14.65
6	1	0	−1	127.70	126.89	118.31	116.82	13.00	12.98	14.51	14.47
7	−1	0	1	356.16	356.97	116.02	117.52	13.00	13.03	14.63	14.68
8	1	0	1	174.94	180.35	130.88	133.01	13.40	13.43	14.93	14.98
9	0	−1	−1	159.09	157.05	99.44	102.83	12.40	12.40	13.99	14.07
10	0	1	−1	166.59	174.84	112.90	113.13	12.60	12.65	14.11	14.10
11	0	−1	1	163.29	155.03	121.60	121.37	12.80	12.75	14.29	14.30
12	0	1	1	181.37	183.41	120.02	116.63	12.60	12.60	14.45	14.37
13	0	0	0	108.18	106.12	121.47	118.19	12.40	12.46	14.05	14.29
14	0	0	0	105.31	106.12	118.35	118.19	12.30	12.46	14.03	14.29
15	0	0	0	107.81	106.12	117.29	118.19	12.60	12.46	14.58	14.29
16	0	0	0	104.30	106.12	116.47	118.19	12.60	12.46	14.55	14.29
17	0	0	0	104.99	106.12	117.38	118.19	12.40	12.46	14.23	14.29

Analysis of variance (ANOVA) was considered to validate the model. Three-dimensional plots were obtained by changing the variables, i.e., one variable constant at the center point and changing the other two variables within the experimental range. Different responses namely viscosity, turbidity, yield, clarity, colour, protein concentration, polyphenol, TSS, and total solid were selected. The model equations with the help of contour plots are used to describe the individual and cumulative effects on the responses.

## Results and discussions

Properties of fresh Jamun juice (with no treatment) are tabulated in Table 1. Response Surface Methodology (RSM) Box–Behnken outline was considered for enhancing the parameter for physico-chemical properties of Jamun juice. Effect of three variables (incubation time, temperature, and concentration) on the exploratory variables, i.e., physical properties (yield, viscosity, turbidity, clarity, and colour) and chemical properties (protein, polyphenol, TSS and total solid) is mentioned in Tables 2 and 3, separately. The analysis of variance (ANOVA) for every reactions demonstrated that the proposed models can clarify more than 90% trial perceptions as a component of independent variables. The proposed model was sufficient with agreeable estimations of *R* ^2^.

The yield of the juice ranges from 73 to 85 ml. Table 2 represents the actual and predicted values of the juice yield. As mentioned in Table 4 temperature (*A*) (*p* < 0.0001) and concentration (*B*) (*p* < 0.0001) have a positive linear effect, whereas time (*C*) (*p* < 0.0001) has a negative linear effect. All the quadratic parameters possess positive effect (*p* < 0.001). The quadratic model obtained for regression analysis for yield in terms of coded levels of variables or the Jamun juice is given below

$$\begin{aligned} {\text{Yield }}\left( \% \right) & = 73.80 \, + \, 0.50A \, + \, 1.25B \, - \, 0.50C \, + \, 0.60A^{2} + \, 3.10B^{2} \\ & \quad + 5.60C^{2} + \, 0.001AB \, - 0.50AC \, + \, 0.001BC. \\ \end{aligned}$$

**Table 4:** Regression coefficients and *R* ^2^ value for all dependent variables for enzymatic clarified Jamun Juice

Regression coefficient	Yield (%)	Viscosity (mPaS)	Turbidity (NTU)	Clarity (%T)	*L*	a*	b*	Protein (mg/g)	Polyphenol (mgGAE/g)	TSS (°B)	Total solid (%)
*b*_0_	73.80	1.10	32.36	29.29	2.31	3.21	−1.21	106.12	118.19	12.46	14.29
*A* (Temp.)	0.50	−4.6 × 10^−3^	14.21	−2.53	−0.21	0.069	0.012	−113.40	5.16	0.050	0.022
*B* (Conc.)	1.25	5.59 × 10^−3^	1.19	−0.83	−0.043	0.018	0.014	11.54	1.39	0.025	0.027
*C* (Time)	−0.50	0.001	0.46	0.64	0.10	−5 × 10^−3^	−0.014	1.64	5.51	0.075	0.13
*A* ^2^	0.06	6.71 × 10^−3^	9.68	17.69	−0.87	−0.95	0.48	131.86	4.42	0.62	0.86
*B* ^2^	3.10	−0.016	−10.12	24.61	−0.71	−1.28	0.49	32.42	−1.84	0.07	0.39
*C* ^2^	5.60	−0.015	−8.46	23.59	−0.69	−1.25	0.55	29.04	−2.86	0.07	−0.47
*AB*	0.011	−7.4 × 10^−3^	−1.81	1.90	0.13	−0.073	−0.051	3.20	−4.14	−0.05	0.099
*AC*	−0.50	3.71 × 10^−3^	0.70	−1.75	−0.025	−0.052	−8.33 × 10^−4^	25.09	2.59	0.15	0.11
*BC*	0.002	3.72 × 10^−3^	1.62	−0.79	0.016	0.037	−0.013	2.65	−3.76	−0.10	9.75 × 10^−3^
*R* ^2^	0.984	0.870	0.99	0.997	0.976	0.999	0.98	0.998	0.9231	0.959	0.941

**Fig. 1:**
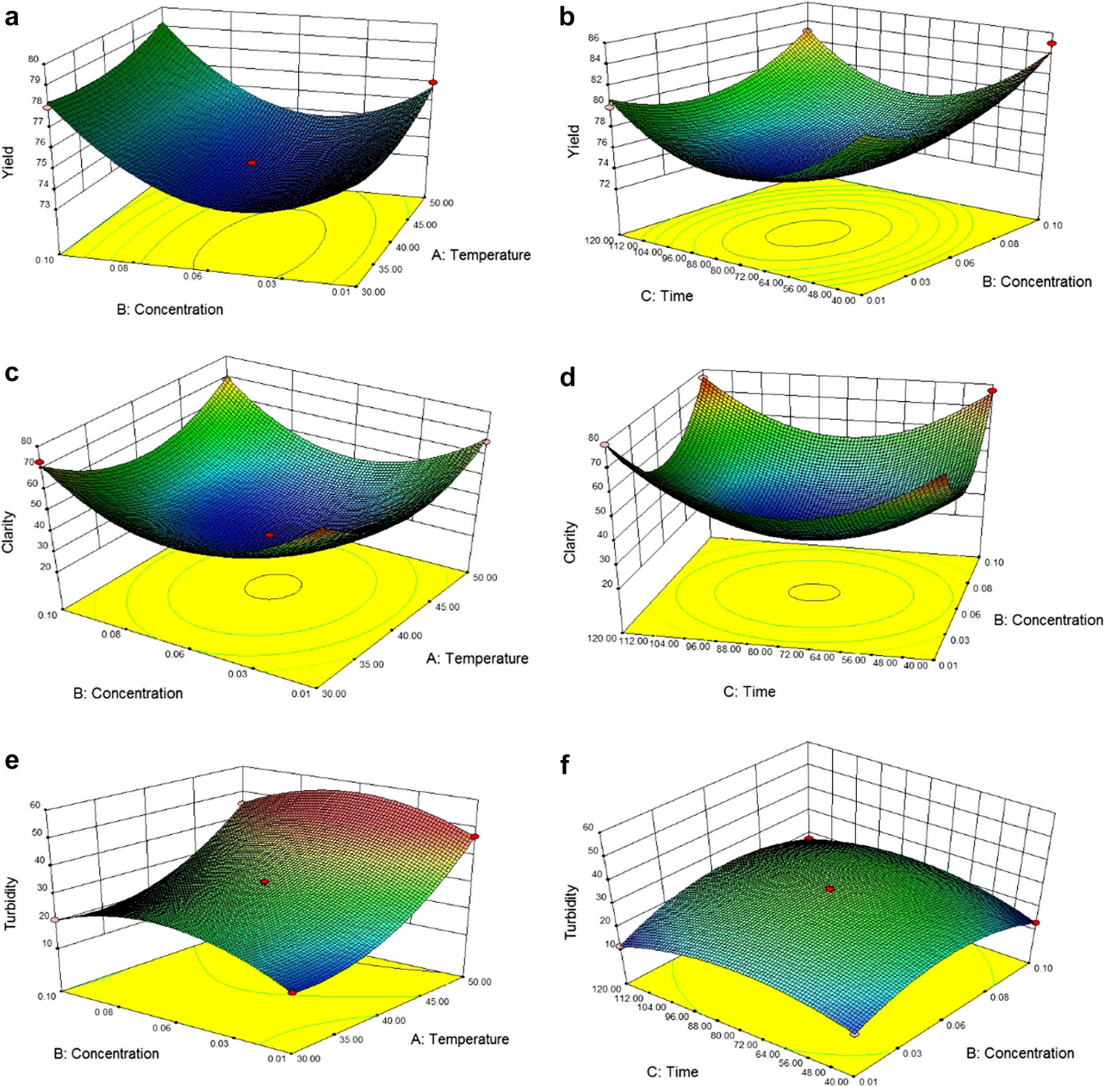
3D Response surface plots for yield, clarity, and turbidity as a function of temperature and enzyme concentration (**a**, **c**, **e**) and enzyme concentration and time (**b**, **d**, **f**)

The fit model was also expressed by coefficient of determination (*R* ^2^) and found to be 98.4% with an adjusted *R* ^2^ value of 96.35%. From the above equation as well as Fig. 1a, it is observed that with increase in concentration of enzyme dosage yield will also increase as it degrades the binding pectinase and protein bonding. Figure 1b represents that the interaction effect of time and concentration has the highest effect on the yield of the Jamun juice. With a highest concentration of 0.1% and nominal time, i.e., 40 min gives the highest yield at 40 °C. Similar trend of data obtained by Surajbhan et al. (2012).

The clarity of the juice ranges from 30 to 80% (%T). Table 2 represents the actual as well as predicted values of clarity of the clarified juice. It can be observed that the juice clarity is minimum at the centre point of experiments. Clarity is one of the most important parameters for the clarified juice. Market trends shows that the maximum the value of the clarity, the more acceptability of the juice by the consumer. From Table 4, it is seen that the temperature (*A*) and enzyme concentration (*B*) have a linear negative effect, whereas time (*C*) as a positive linear effect. All the quadratic effects are positive in nature, but interaction terms have a negative effect. The regression equation for clarity of juice with respect to temperature (*A*), concentration (*B*), and time (*C*) in terms of their real value is given as follows:

$$\begin{aligned} {\text{Clarity }}\left( {\% \, T} \right) & = \, 29.29 - 2.53A \, + \, 0.83B + 0.64C + 17.69A^{2} + 24.61B^{2} \\ & \quad + \, 23.59C^{2} + \, 1.9AB - 1.75AC - 0.79BC. \\ \end{aligned}$$

The coefficient of determination (*R* ^2^) for the above equation is 0.99 which implies that the regression model can analyse 99% of data variability. The same trend has been followed where changes in the interaction affect temperature—enzyme concentration Fig. 1c and time—temperature first decrease the clarity and then increase. Figure 1d shows the time–concentration interaction helped to obtain the maximum clarity as with the increasing concentration. At a certain temperature when the time increases, the clarification of juices will be more than the less time. At a lower concentration of enzyme dosage and maximum time, clarity is maximum. Similar trend has been noticed by Lee et al. (2006) and Shah and Nath (2007) for clarification of banana and litchi juice, respectively. Whereas Abdullah et al. (2007) observed a different trend in clarity of carambola juice.

Turbidity ranges from 12 to 47 NTU. Turbidity is considered as one of the major parameter for the clarification process. The lower the value of the turbidity, the acceptability will be more. From Table 4, it is clearly observed that three factors temperature (*A*) (*p* < 0.0001), enzyme concentration (*B*) (*p* < 0.0001), and time (*C*) (*p* < 0.0001) all have a positive linear effect, but in case of quadratic terms except temperature (*p* < 0.001), concentration and time have negative effect (*p* < 0.001). The regression equation for turbidity with respect to temperature (*A*), concentration (*B*), and time (*C*) can be represented as:

$$\begin{aligned} {\text{Turbidity }}\left( {\% {\text{ NTU}}} \right) &= { 32}. 3 6 + { 14}. 2 1 {A} + { 1}. 1 9 {B} \\ &\quad + 0. 4 6 {C} + { 9}. 6 8 {A}^{ 2} {-}{ 1}0. 1 2 {B}^{ 2} {-}{ 8}. 4 6 {C}^{ 2} \\ &\quad - { 1}. 8 1 {AB} + \, 0. 70{AC} + { 1}. 6 3 {BC} . \end{aligned}$$

The interaction term has no significant value. The *R* ^2^ value for the above equation is 0.99 which implies that the model can analyse 99% of data variability. From Fig. 1e, it is observed that positive slope is obtained with the interaction effect of concentration and temperature. With the increase in temperature and concentration, turbidity also increases. In case of Fig. 1f, interaction effect of time and concentration has a different trend. With respect to time as the concentration of enzyme increased, turbidity value also increases, but after a certain period, it started decreasing. Similar trend had obtained by Abdullah et al. (2007) and Lee et al. (2006) for clarification of carambola juice and banana juice with a positive linear coefficient and negative quadratic coefficients. Pinelo et al. (2010) descried that the turbidity of the cherry juice clarification has a similar trend with the values of the recent data. The results for clarity and turbidity are supporting the data with each other. From the centre point, clarity is low which represents that turbidity will be high at that point.

It is seen from Table 4 that the viscosity was affected by all the three parameters, such as temperature (*A*) (*p* < 0.0001) has a negative linear effect, and concentration (*B*) (*p* < 0.001) and time (*C*) (*p* < 0.001) have a positive linear effect. Quadratic effect is positive in case of temperature. The regression model for viscosity with respect to temperature (*A*), concentration (*B*), and time (*C*) can be represented as:

$$\begin{aligned} {\text{Viscosity }}\left( {\text{mPaS}} \right) & = \, 1.1 - 4.6 \times 10^{ - 3} A + 5.59 \times 10^{ - 3} B + 0.001C + 6.71 \times 10^{ - 3} A^{2} - 0.016B^{2} - 0.015C^{2} \\ & \quad - 7.40 \times 10^{ - 3} AB + 3.71 \times 10^{ - 3} AC + 3.72 \times 10^{ - 3} BC. \\ \end{aligned}$$

**Fig. 2:**
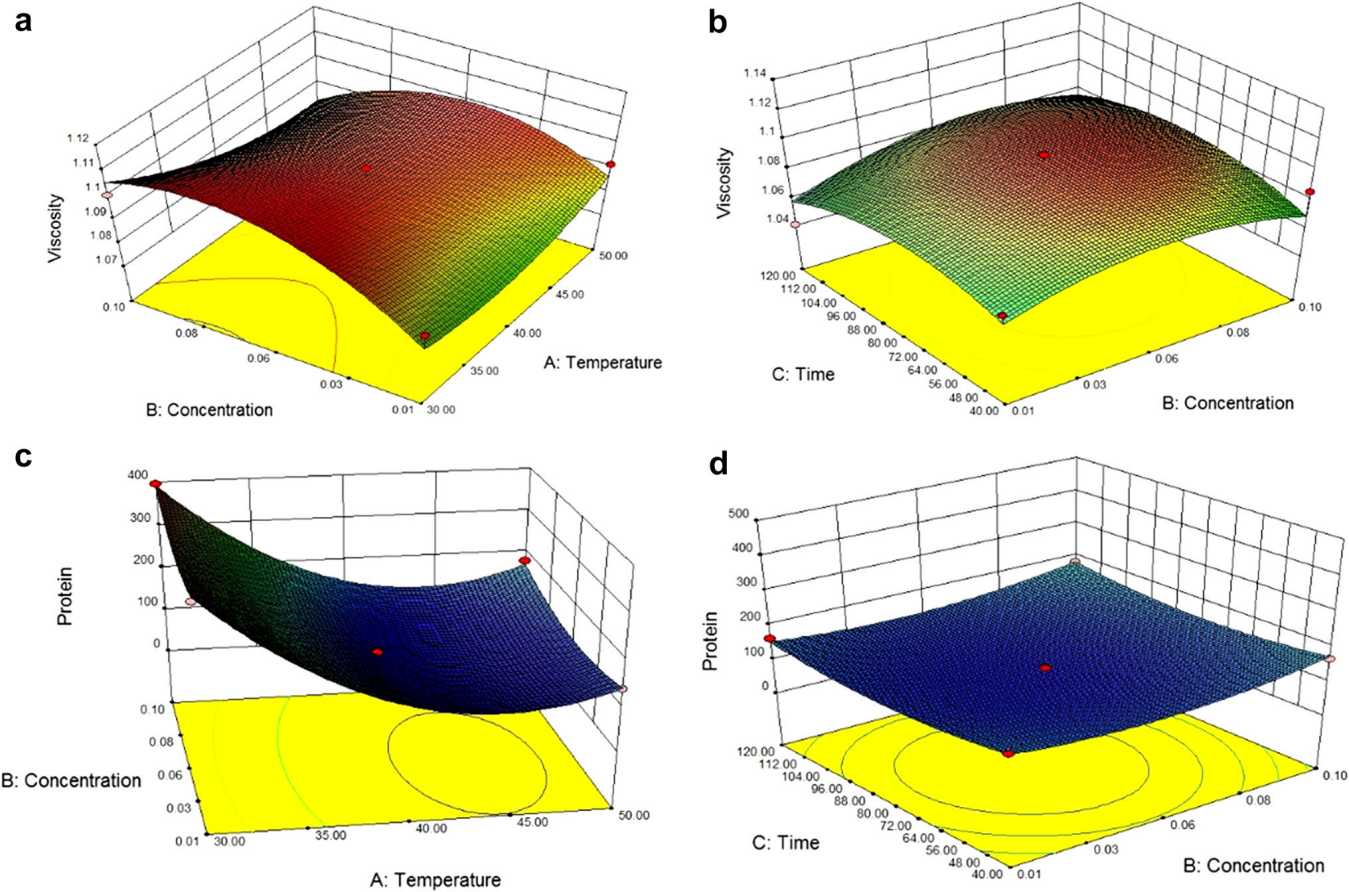
3D Response surface plots for viscosity and protein as a function of temperature and enzyme concentration (**a**, **c**) and enzyme concentration and time (**b**, **d**)

The *R* ^2^ value for the above equation is 0.87 which implies that the regression model can analyse 87% of data variability for viscosity. Figure 2a represents that as temperature and concentration decreases, viscosity decreases and become constant at its maximum level. Again from Fig. 2a, it is seen that enzyme concentration is directly proportional to viscosity. Pectin forms gel structure which increases the viscosity at high temperature. However, at temperature between 30 and 40 °C, the water holding capacity of pectin is reduced which results in lower viscosity (Kittur et al. 2003). Figure 2b gave a perfect concave nature of the graph which implies that concentration of the enzyme has the main effect on the viscosity of the juice.

Colour is divided into three different parameters where L* value represents the lightness and darkness of the juice, positive a* value represents the redness and negative b* value represents the blueness of the juice. The negative the colour of the b* value characteristics, colour of the jamun juice can be obtained. From Table 4, in case of L*, temperature (*A*) (*p* < 0.0001) and concentration (*B*) (*p* < 0.0001) have a negative linear and quadratic value, whereas interaction terms (*p* < 0.05) are positive in nature. For a* value, temperature (*A*) (*p* < 0.0001) and concentration (B) (*p* < 0.0001) have a positive value, but time (C) (*p* < 0.0001) has a negative slope. The quadratic values for all the parameter (*p* < 0.001) possess negative value. In case of b* value, temperature (*A*) (*p* < 0.0001) and concentration (*B*) (*p* < 0.0001) have a positive linear effect, but time (*C*) (*p* < 0.0001) has a negative linear effect. All the quadratic effects (*p* < 0.001) are positive, but the interaction terms are (*p* < 0.05) are negative in nature. The regression model for colour is given as

$$\begin{aligned} L* \, & = \, 2.31 - 0.20A - 0.043B + 0.01C - 0.87A^{2} \\ &\quad - 0.71B^{2} - 0.69C^{2} + 0.13AB - 0.02AC + 0.015BC \\ a* \, & = \, 3.21 + 0.069A + 0.018B{-}5 \times 10^{ - 3} C - 0.95A^{2} \\ &\quad - 1.28B^{2} - 1.25C^{2} - 0.073AB - 0.052AC - 0.037BC \\ b* \, & = \, - 1.21 + 0.012A + 0.014B{-}0.014C + 0.48A^{2} \\ &\quad + 0.49B^{2} + 0.55C^{2} - 0.051AB - 8.33 \times 10^{ - 4} AC \\ &\quad - 0.013BC. \\ \end{aligned}$$

**Fig. 3:**
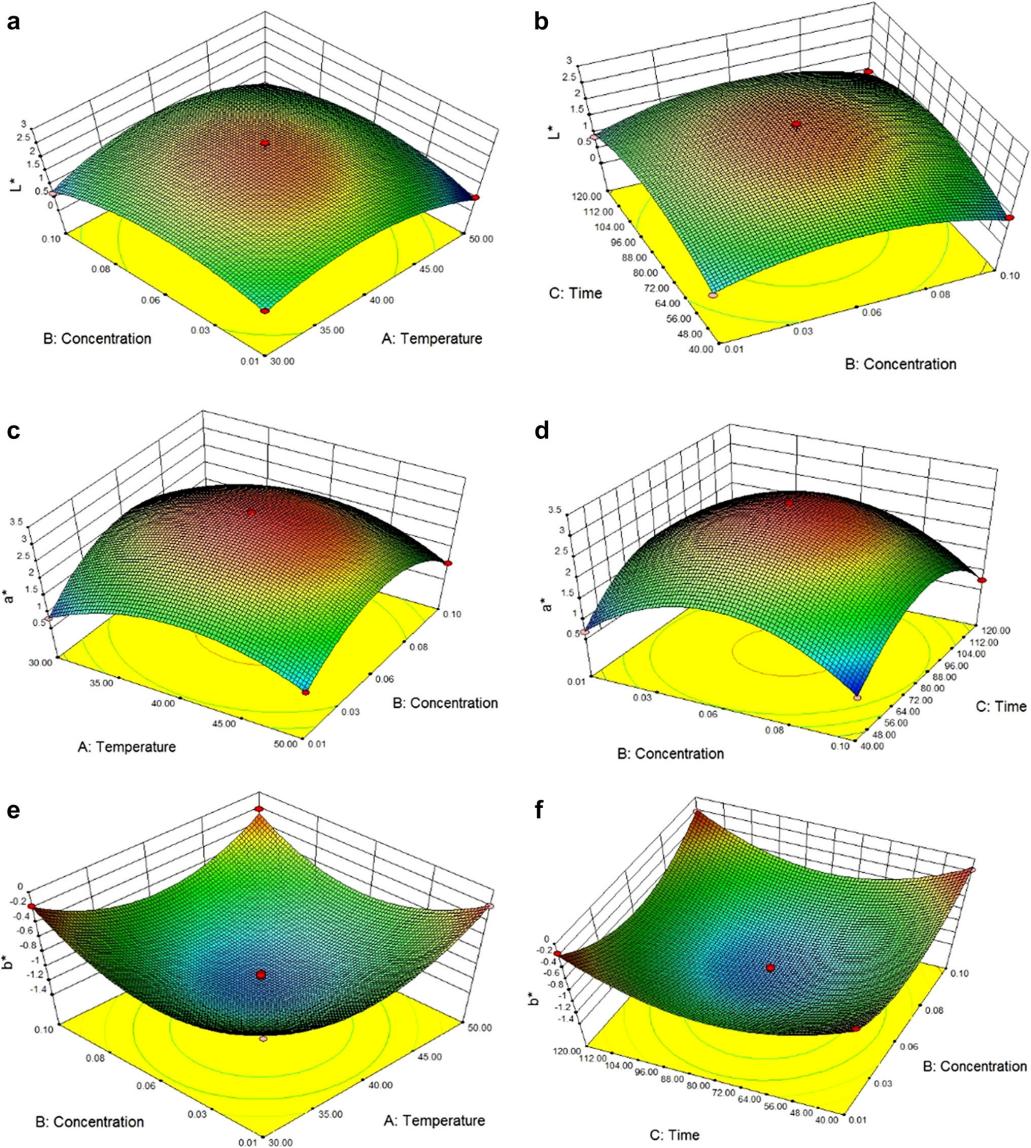
3D Response surface plots for colour values (L*, a*, b*) as a function of temperature and enzyme concentration (**a**, **c**, **e**) and enzyme concentration and time (**b**, **d**, **f**)

The *R* ^2^ value for L*, a*, and b* equations is 0.97, 0.99, and 0.98, respectively which implies that the model can analyse 97, 99, and 98% of data variability. From Fig. 3a, as the increase in concentration and temperature, there is no significant changes in value, but in a combined effect, L* value decreases significantly. The same trend is observed from Fig. 3b, so it can be concluded that time has a positive trend which can increase lightness of the clarified juice. This result can be justified by the results obtained from the clarity values. Similar trend in the L* value is obtained by Abdullah et al. (2007). In case of a* value from Fig. 3c, as the temperature and concentration increased at first, the value of a* will increase and then it hinders. With the combination of both the value decreases. The highest value for the redness obtained at the centre point with a value of 3.24. For b* value Fig. 3e, f shows that with respect of independent variables, the value decreases up to 0.06% concentration, 40 °C temperature, and 80 min, and then the value shoots up. The more negative the value, the original colour of Jamun juice can be obtained. The most negative value obtained at the centre point with −1.4.

Denaturation of protein–pectin bond is one of the major process mechanisms for the clarification of the juice. Increase of the amount of protein in the clarified juice signifies a proper clarification process. Ranges of protein content is in between 105 and 409 mg/g. Table 4 signifies that only temperature (*A*) (*p* < 0.0001) has a linear negative effect, whereas time (*C*) and enzyme concentration (*B*) (*p* < 0.0001) have positive linear slope. The quadratic terms (*p* < 0.001) also have a positive effect with a positive interaction effect. The regression model representing the effect of temperature, enzymatic concentration, and time on Jamun juice in terms of their real value is given as

$$\begin{aligned} {\text{Protein}}\left( {{\text{mg}}/{\text{g}}} \right) & = 106.12{-}113.4A + 11.54B{-}1.64C \\ &\quad {-}131.86A^{2} + 32.42B^{2} - 29.04C^{2} \\ & \quad {-}3.20AB + 25.09AC - 2.65BC. \\ \end{aligned}$$

The coefficient of determination (*R* ^2^) for the fit model was determined as 0.99 which indicates that the regression model is able to explain 99% of the variability of the data. Figure 2c shows that as the temperature increases, amount of protein decreases but when the concentration of enzymatic dosage goes up amount of protein content increases. As the enzyme used in this study obtained from a strain, *Aspergillus aculeatus* has a particular effective time zone where it can give its best result. Figure 2d shows that time has a positive slope but not significant. Therefore, it can be concluded that concentration of the enzyme has the maximum effect on the protein amount and then time also has a significant role. At lowest temperature 30 °C with 0.06% concentration with 40 min treatment time, maximum retention of the protein content 409.30 mg/g had obtained.

Phenol is one of the major components in Jamun with the effect of enzyme concentration and time–temperature effect. Extraction of phenol content increases in the clarified juice. As the clarification process varies with the parameters, the amount of polyphenol ranges between 99 and 130 mgGAE/g. In case of polyphenol, all the independent parameter temperature (*A*) (*p* < 0.0001), enzyme concentration (*B*), and time (*C*) (*p* < 0.0001) have positive linear effect with a negative quadratic effect for enzyme concentration (*B*) and time (*C*) (*p* < 0.001). The interaction effects are also negative (*p* < 0.05). The regression model for polyphenol with the coded value can be represented as

$$\begin{aligned} &{\text{Polyphenol}}\left( {{\text{mg GAE}}/{\text{g}}} \right)\\ &\quad = 118.19 + 5.16A + 1.39B \\ &\qquad + 5.51C + 4.42A^{2} - 1.84B^{2} \\ & \qquad - 2.8C^{2} - 4.14AB + 2.59AC - 3.76BC. \\ \end{aligned}$$

**Fig. 4:**
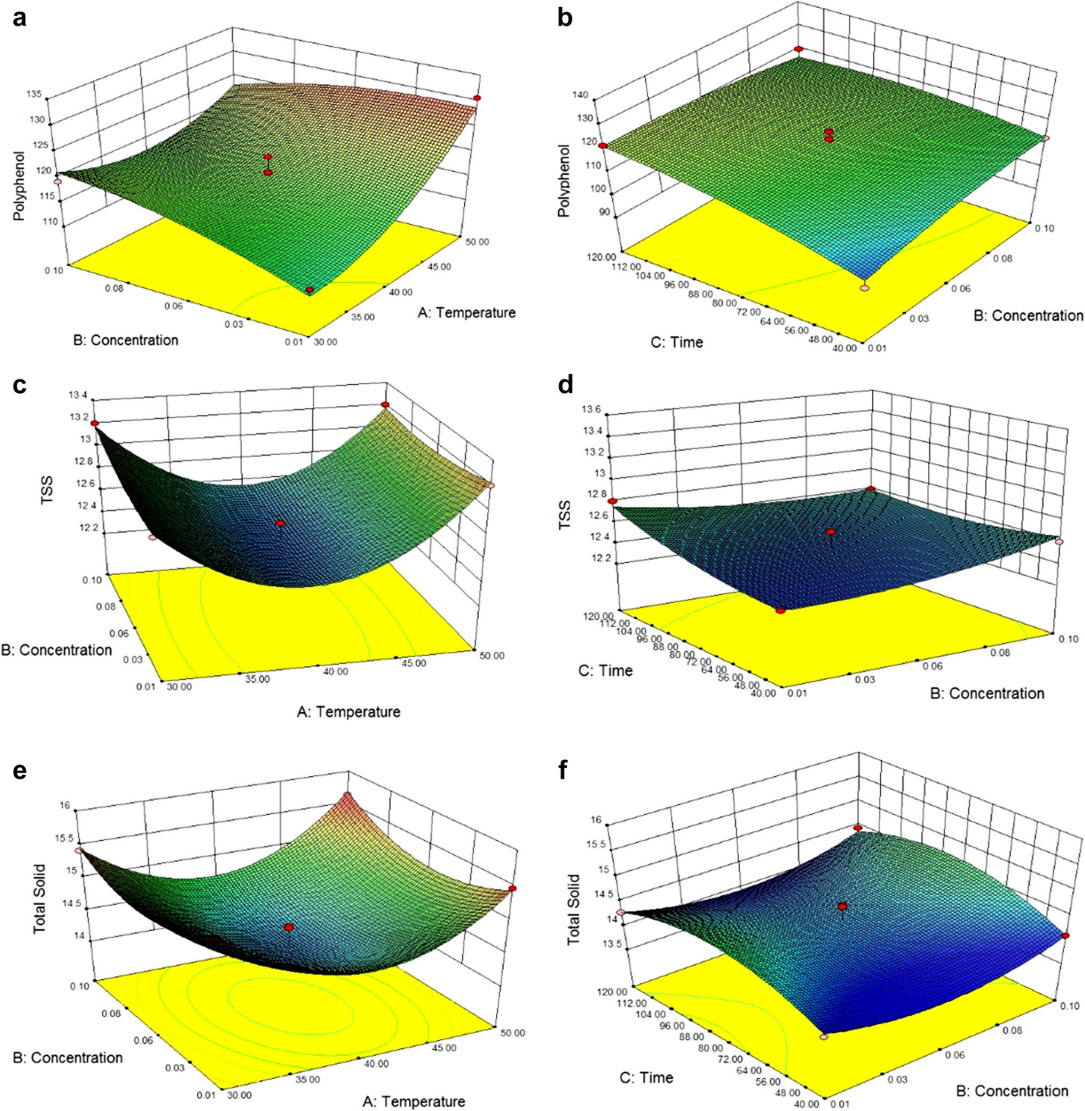
3D Response surface plots for polyphenol, TSS, and total solid as a function of temperature and enzyme concentration (**a**, **c**, **e**) and enzyme concentration and time (**b**, **d**, **f**)

The values for coefficient of determination (*R* ^2^) are 0.92, which indicates that the regression model is able to explain 92% of variability of the polyphenol concentration of the Jamun juice. Figure 4a shows that enhancing of concentration and temperature will also enhance the polyphenol value, but in combined interaction, the value increases up to a level and then follows the decreasing trend. From Fig. 4b, combined effect of enzyme concentration and time has negative effect. Increasing of time and temperature will increase the polyphenol content and interaction effect will increase the polyphenol value at its maximum which signifies the coefficient of interaction value of *A* and *C* is positive. Therefore, it can be conclude that time–temperature effect is playing the major role in case of polyphenol activities. At 50 °C with 0.06% concentration and 120 min incubation time, highest polyphenol content can be obtained 130.8 mgGAE/g.

Total soluble solid or sugar present in the juice will be another important nutritional parameter for the clarified process. Table 4 clearly shows that temperature (*A*), enzyme concentration (*B*), and time (*C*) (*p* < 0.0001) have positive linear effect with a positive quadratic effect. The interaction effects are negative (*p* < 0.05) at significant level. The regression model for TSS with the coded value can be represented as

$$\begin{aligned} {\text{TSS }}\left( {^{ \circ } {\text{B}}} \right) &= 12.46 + 0.05A + 0.025B + 0.075C + 0.62A^{2} \\ & \quad + 0.07B^{2} + 0.71C^{2} - 0.05AB + 0.15AC - 0.10BC. \\ \end{aligned}$$

The values for coefficient of determination (*R* ^2^) are 0.95, which indicates that the model can explain 95% of variability of the TSS of the Jamun juice. From Fig. 4c, a perfect convex quadratic curve has obtained where increase in concentration enhances the values of the TSS, but temperature acts first in a decreasing trend, then it increases up. From Fig. 4d, it can conclude that time has a positive inclination on TSS values, whereas interaction effect of time and concentration of enzyme has almost a linear trend. Concentration and temperature play the major roles in changing the TSS value. At a temperature of 40 °C with 0.01% concentration, TSS value is minimum 12.4 °B.

Clarified juice should contain less amount of total solid. Therefore, measurement of total solid can quantify the process of clarification. The ranges of total solid content in the experimental setup were 13.9–15.6%. From Table 4, it is clearly visible that temperature (*A*), enzyme concentration (*B*), and time (*C*) (*p* < 0.0001) have positive linear effect, and in case of quadratic effect, time (*C*) (*p* < 0.0001) has negative trend. Interaction effects also have positive impact. The regression model for total solid with the coded value can be represent as

$$\begin{aligned} &{\text{Total solid }}\left( \% \right) \\ &\quad = 14.29 + 0.022A + 0.027B + 0.13C + 0.86A^{2} + 0.39B^{2} \\ & \qquad - 0.47C^{2} + 0.099AB + 0.11AC + 9.75 \times 10^{ - 3} BC. \\ \end{aligned}$$

The coefficient of determination (*R* ^2^) is 0.94, which indicates that the model can explain 94% of variability of the total solid content of the Jamun juice. From Fig. 4e, it can be said that concentration of enzyme has a direct positive approach where in case of temperature, total solid value decreases up to 40 °C and then it increases again. As the time increases, the value of TS enhances, but interaction of time–temperature combination decreases the total solid in the mid ranges. Coagulation of protein polyphenol takes place at this time. From Fig. 4f, it can be concluded that interaction effect of time and concentration has a negative approach to the TS. The least value for the TS (13.9%) obtained at 40 °C with 0.01% concentration at 40 min of time.

## Conclusion

The optimum conditions for clarification and extractions were determined on the basis of high yield percent, L* value, a* value, protein, and polyphenol content and lower values for turbidity, viscosity, clarity, b* value, TSS, and total solid content. According to the optimised-dependent parameters, the incubation temperature should be 40 °C with 80 min and the concentration of enzyme will be 0.05% (w/v). At the optimised condition, the value of major parameters was turbidity: 32.21 NTU; clarity: 74.39% T; viscosity: 1.07 mPaS; polyphenol: 115.31 mg GAE/g; protein: 102.43 mg/g; yield: 75%; TSS: 12.4 °B; total solid: 13.97%; L*: 2.34; a*: 3.25, b*: −1.30. For the clarification process, 3D contour plots with the help of RSM signify better understanding of the results.
